# Proteomic Analysis of Renal Biomarkers of Kidney Allograft Fibrosis—A Study in Renal Transplant Patients

**DOI:** 10.3390/ijms21072371

**Published:** 2020-03-30

**Authors:** Line Aas Mortensen, Anne Marie Svane, Mark Burton, Claus Bistrup, Helle Charlotte Thiesson, Niels Marcussen, Hans Christian Beck

**Affiliations:** 1 Department of Nephrology, Odense University Hospital, DK-5000 Odense, Denmark; line.mortensen@rsyd.dk (L.A.M.); claus.bistrup@rsyd.dk (C.B.); helle.thiesson@rsyd.dk (H.C.T.); 2 Department of Epidemiology, Biostatistics and Biodemography, University of Southern Denmark, DK-5000 Odense, Denmark; annemarie@math.aau.dk; 3 Department of Clinical Genetics, Odense University Hospital, DK-5000 Odense, Denmark; mark.burton@rsyd.dk; 4 Department of Pathology, Odense University Hospital, DK-5000 Odense, Denmark; niels.marcussen@rsyd.dk; 5 Department of Clinical Biochemistry and Pharmacology, Centre for Clinical Proteomics, Odense University Hospital, DK-5000 Odense, Denmark

**Keywords:** renal transplantation, interstitial fibrosis and tubular atrophy, IFTA, proteomics

## Abstract

Renal transplantation is the preferred treatment of end stage renal disease, but allograft survival is limited by the development of interstitial fibrosis and tubular atrophy in response to various stimuli. Much effort has been put into identifying new protein markers of fibrosis to support the diagnosis. In the present work, we performed an in-depth quantitative proteomics analysis of allograft biopsies from 31 prevalent renal transplant patients and correlated the quantified proteins with the volume fraction of fibrosis as determined by a morphometric method. Linear regression analysis identified four proteins that were highly associated with the degree of interstitial fibrosis, namely Coagulation Factor XIII A chain (estimate 18.7, adjusted *p* < 0.03), Uridine Phosphorylase 1 (estimate 19.4, adjusted *p* < 0.001), Actin-related protein 2/3 subunit 2 (estimate 34.2, adjusted *p* < 0.05) and Cytochrome C Oxidase Assembly Factor 6 homolog (estimate −44.9, adjusted *p* < 0.002), even after multiple testing. Proteins that were negatively associated with fibrosis (*p* < 0.005) were primarily related to normal metabolic processes and respiration, whereas proteins that were positively associated with fibrosis (*p* < 0.005) were involved in catabolic processes, cytoskeleton organization and the immune response. The identified proteins may be candidates for further validation with regards to renal fibrosis. The results support the notion that cytoskeleton organization and immune responses are prevalent processes in renal allograft fibrosis.

## 1. Introduction

Kidney transplantation is the preferred treatment of end stage renal disease, reducing mortality when compared to any type of dialysis [1], and increasing quality of life [2]. The introduction of modern induction therapy, as well as highly effective immunosuppressive regimens, has reduced graft loss due to acute rejection [3]. Over time, however, kidney graft function inevitably deteriorates. Improving long-term graft survival remains a key issue in renal transplantation.

The histological finding of chronic allograft lesions with no known etiology is referred to as IFTA (interstitial fibrosis and tubular atrophy) [4]. IFTA is often accompanied by deteriorating renal function, and the presence of IFTA predicts an adverse renal outcome [5]. The development of kidney fibrosis is a multifactorial process including inflammation and ischemia, which ultimately leads to the deposition of extracellular matrix proteins [6].

The causes of chronic allograft failure are diverse and might include acute or chronic rejection, recurrent disease, drug toxicity or infection [7]. Irrespective of the primary insult, IFTA is characterized by largely irreversible damage. Hence, IFTA is important to distinguish from reversible conditions that need specific interventions. Also, insight into the pathogenesis of IFTA might provide the opportunity for early, preventative therapy and guide the development of pharmacological interventions.

Currently, the diagnosis of IFTA is made by kidney biopsy. Although a minor procedure, a biopsy introduces a risk of a gross hematuria of 3%–4%, and a risk of a perirenal hematoma of 2%–4% [8], which necessitates post-procedural observation. Furthermore, there is a risk of sampling variability [9]. Much effort has been devoted to identifying biomarkers to guide diagnostics in acute or chronic renal failure, but so far, no biomarkers are in routine clinical use. In tissue, proteins are prevalent in both the intracellular and extracellular compartments, and have diverse functions, serving as structural components and as mediators of a wide range of biological functions (i.e., as transcription factors, hormones, antibodies and enzymes) [10]. The emergence of large-scale proteomic approaches provides a unique opportunity to gain insight into proteins involved in specific diseases. The use of proteomics is, however, critically dependent on interpreting results in context to make biological sense of the large amounts of information provided [11].

Only a few studies have investigated the proteome in chronic allograft dysfunction. Early discovery-driven proteogenomic analyses of biopsies with varying degrees of IFTA revealed several differences in the proteome between fibrotic and non-fibrotic kidneys [12]. The objective of the current study was to identify proteins that correlate with the degree of fibrosis in renal biopsies from stable kidney transplant patients. The purpose was to gain insight into the mechanisms of fibrogenesis and to identify biomarker candidates to guide the diagnosis of IFTA. To address this, a set of biopsies from 31 prevalent renal transplant patients were evaluated by explorative in-depth proteomics based on nano-liquid chromatography combined with orbitrap mass spectrometry analysis and 10-plex tandem mass tags.

## 2. Results

Biopsies from 31 individuals were examined and their clinical characteristics are summarized in Table 1. Twenty two (71%) participants were male and the median age was 60 (ranging from 24–73) years. The median time since transplantation was 3 years, but ranged from 0.3 to 12.8 years. All patients were treated with a calcineurin inhibitor. The most prevalent immunosuppressive regimen was tacrolimus in combination with mycophenolate mofetil, but four subjects (13%) were additionally treated with a small dosage of prednisolone. The majority of the included subjects had hypertension (94%). Two patients (6%) had diabetes at the time of inclusion. Three patients had a previous rejection occurring between 2 and 3.5 years prior to inclusion in the study. In all cases, the rejection episode was treated with prednisolone, and renal function returned to pre-rejection levels. The fibrosis quantifications in these subjects at inclusion were below average, at 28%, 29% and 32%, respectively.

### 2.1. Characterization of Fibrosis in Kidney Allograft Biopsies

**Figure 1:**
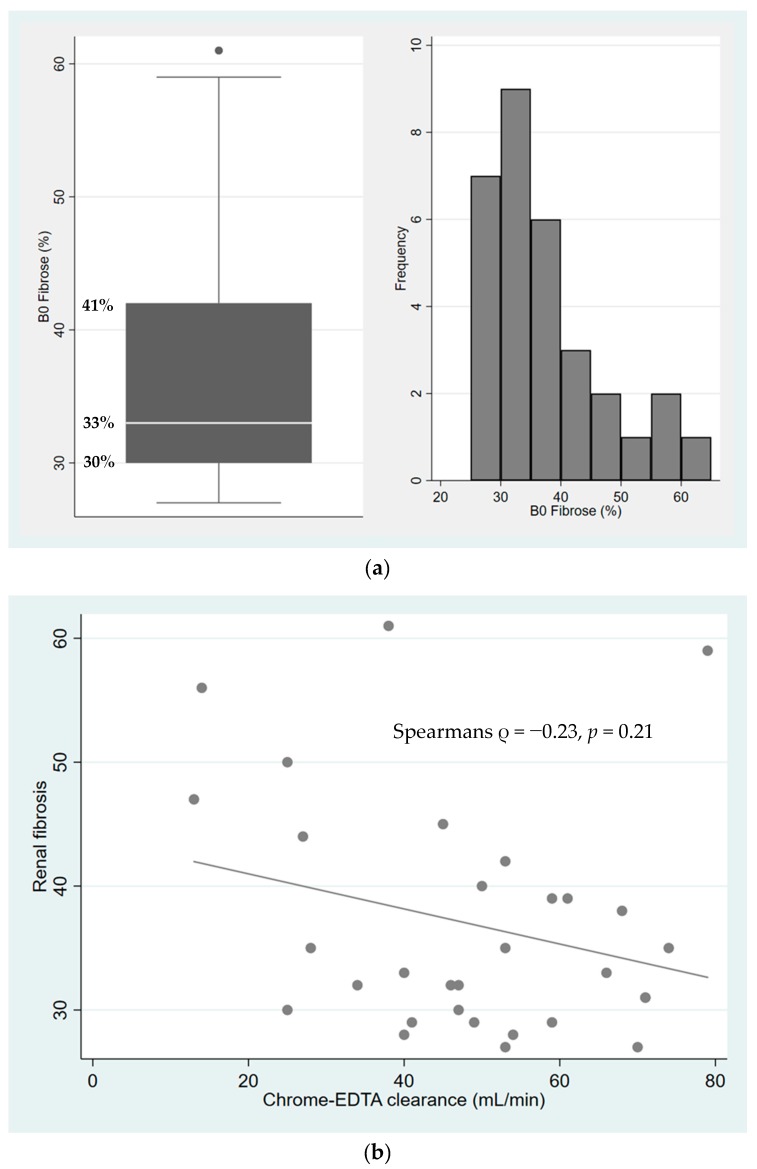
(**a**) The distribution of interstitial fibrosis by point counting, whiskers indicate upper and lower adjacant values, and dot represent an outlier above the upper adjacant value; (**b**) The association between renal function and the degree of fibrosis (Spearmans ρ = −0.23, *p* = 0.21).

The distribution of interstitial fibrosis was evaluated by point counting (Figure 1a) using the Banff Lesion Score method, which relies on the assessment of the presence and degree of different histopathological changes in different compartments of the renal biopsy, such as fibrosis, interstitial inflammation, and mild tubulitis, important for the diagnosis of graft rejection. The median number of points counted per biopsy was 284 (IQR 252–329). The median extent of fibrosis, estimated as the volume fraction, was 33% (IQR 30%–41%). Interstitial inflammation was present in 16 biopsies (*n* = 12 (29%) for Banff I1 score, *n* = 3 (10%) for Banff I2 score and *n* = 1 (3%) for Banff I3 score). Mild tubulitis (Banff T1 score) was present in three biopsies (10%) (see Supplementary Table S1 for a complete summary of the Banff scores). No biopsies gave the suspicion of acute or chronic rejection.

The association between renal function and the extent of fibrosis is shown in Figure 1b. The variation in the extent of fibrosis increased with decreasing renal function. There was a weak, but not significant, correlation between renal function and the volume fraction of interstitial fibrosis (Spearman’s ρ = −0.23, *p* = 0.21).

### 2.2. Kidney Allograft Proteomics

Nano-LC-MSMS analysis analyzed 4717 proteins across the analyzed biopsies, whereof 1973 proteins were quantified in all patient biopsies (Supplementary Table S2). Having missing values is a well-known phenomenon in quantitative mass spectrometry-based proteomics when using data-dependent acquisition, and we found that the number of missing values per quantified protein acceptable for downstream statistical analyses was 11, leaving a dataset of 2687 proteins analyzed in 31 kidney biopsies for further evaluation.

### 2.3. Proteins Correlated with the Degree of Fibrosis

**Figure 2:**
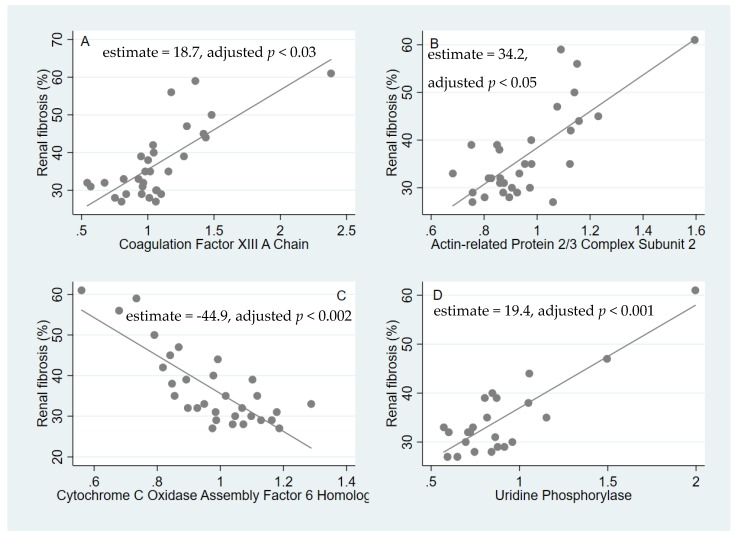
The associations of specific proteins with the degree of fibrosis. The x-axis shows the relative protein abundances (**A**–**D**).

The multiple linear regression identified 26 proteins with an adjusted p-value of less than 0.5 (Table 2). Interestingly, four proteins were highly significantly associated with the degree of fibrosis after correction for multiple testing. Of these, three were positively associated: Coagulation Factor XIII A chain (estimate = 18.7, adjusted *p* < 0.03, Figure 2a), Uridine Phosphorylase 1 (estimate = 19.4, adjusted *p* < 0.001, Figure 2d) and Actin-related protein 2/3 subunit 2 (estimate = 34.2, adjusted *p* < 0.05, Figure 2b). Cytochrome C Oxidase Assembly Factor 6 homolog was negatively associated (estimate = −44.9, adjusted *p* < 0.002, Figure 2c).

Then we performed a LASSO analysis in order to identify the most predictive proteins for fibrosis (Supplementary Table S3). The nine proteins selected by the LASSO in at least 10% of the subsamples are listed in Table 3. 

Interestingly, Cytochrome C Oxidase Assembly Factor 6 homolog (selection probability 56.7%), Coagulation Factor XIII A chain (selection probability 21.7%) and Actin-related protein 2/3 subunit 2 (selection probability 10.7%), which all strongly correlated with the degree of fibroses, were also highly predictive of fibrosis. The fourth protein that strongly correlated with fibrosis, Uridine Phosphorylase 1, was not identified in the analysis, most probably because this protein was not detected in all biopsies, and only proteins with no missing values across patients were included in the LASSO analysis. 

In order to identify molecular pathways and biological processes, we evaluated 133 proteins positively correlated with fibrosis (unadjusted *p* < 0.005) by Gene Ontology enrichment analysis. This analysis revealed an abundance of proteins mainly related to catabolic processes, cytoskeleton organization and immune responses (Table 4). By contrast, the 76 proteins negatively associated with fibrosis (unadjusted *p* < 0.005) were mainly involved in normal metabolic processes and respiration (Table 4 and Supplementary Table S4).

## 3. Discussion

The current study investigated kidney graft biopsies from patients with creatinine clearance ≥ 30 mL/min and varying degrees of interstitial fibrosis by in-depth quantitative proteomic analysis using nano-LC-MSMS combined with 10-plex tandem mass tags (TMT), and the current study constitutes a first step towards the identification of non-invasive biomarkers of renal allograft fibrosis. Our proteomic analysis of renal tissue provides a detailed characterization of the protein composition of renal allografts; however, proteins of very low abundance may not be detected. Despite this, we identified four proteins—Coagulation Factor XIII A chain, Actin-related protein 2/3 subunit 2, Cytochrome C Oxidase Assembly Factor 6 homolog and Uridine Phosphorylase 1—that, even after multiple testing, showed a strong correlation with renal allograft fibrosis. Moreover, three of these proteins were also shown to be strongly predictive for fibrosis.

Coagulation factor XIII (FXIII) A chain was positively correlated with the degree of fibrosis and was also chosen by the LASSO in 21.7% of cases. FXIII is synthesized in cells of bone-marrow origin (i.e., thrombocytes and monocytes) [13]. Circulating FXIII is a tetramer consisting of two A chains and two B chains. The A chain can be activated by thrombin and calcium to become a transglutaminase that cross-links and stabilizes fibrin, thereby catalyzing the final steps in the coagulation cascade [14]. FXIII also cross-links proteins of the extracellular matrix [15], increases the proliferation of monocytes, and further increases the migration and decreases the apoptosis of both monocytes and fibroblasts [16]. While the recruitment and longevity of fibroblasts and the cross-linking of ECM proteins are beneficial in the context of wound healing, they constitute core processes in the progression of tissue fibrosis. Thus, it is feasible that the overexpression of FXIII might serve to promote fibrogenesis through increased fibroblast activity and inflammation, and reduced ECM degradation.

Actin-related protein 2/3 (ARP2/3) subunit 2 was similarly strongly associated with fibrosis. ARP2/3 is a protein complex involved in actin cytoskeleton organization and has been associated with a range of cell functions, primarily relating to cell motility [17]. Of possible interest in relation to fibrosis is the observation that disrupting actin cytoskeleton assembly via ARP2/3 in fibroblasts reduces fibroblast motility in an in vitro model of wound healing, presumably by preventing the polarization of the Golgi apparatus [18]. A high renal abundance of ARP2/3 might be an indicator of ongoing inflammation or increased fibroblast motility.

Cytochrome C oxidase is located in the mitochondrial membrane and plays a major role in cell respiration and ATP synthesis in eukaryotic cells [19]. Cytochrome C oxidase assembly factor, also located in the mitochondrion, contributes to the correct assembly and function of Cytochrome C oxidase [20]. Cytochrome C oxidase assembly factor was negatively correlated to fibrosis and chosen by the LASSO in 56.7% of cases, which indicates that it is a strong predictor of fibrosis. This observation may reflect a lack of healthy cells in the fibrotic kidneys. A proteomic study of CsA toxicity to human proximal tubular cells in vitro found a reduction of proteins from the inner mitochondrial membrane associated with CsA treatment [21]. One obvious difference to the current study, however, was the use of cells rather than renal biopsies. The proteomic analysis of biopsy specimens includes all parts of the nephron, as well as invading cells and fibrotic areas, which constitute a more heterogeneous sample than a single cell line.

Uridine phosphorylase catalyzes the reversible conversion of uridine to uracil, which is an important step in the pyrimidine salvage pathway [22]. We found a positive correlation between this protein and the extent of fibrosis. The previously mentioned in vitro study of human proximal tubule cells treated with CsA found an upregulation of proteins associated with purine salvage in relation to CsA treatment. The authors hypothesized that respiratory chain dysfunction led the cells to use this pathway rather than the more energy-consuming de novo synthesis [21]. Uridine phosphorylase is present in most human cells and can be induced by inflammatory cytokines in a number of tumor cell lines [23]. It has previously been shown to co-localize with intermediate filament vimentin in fibroblasts and colon cells; however, the significance of this co-localization is unknown [22].

Functional annotation of the proteins most significantly associated with fibrosis and eGFR revealed a marked difference between those positively and those negatively associated, i.e., proteins positively associated with fibrosis were negatively associated with eGFR and vice versa, indicating that kidney function and fibrosis are linked processes at the proteome microenvironment level (Table 4 and Supplementary Table S5). The observation that proteins involved in cytoskeleton organization and immune responses were abundant in fibrotic tissue has been reported in previous studies.

Nakorchevsky et al. performed a proteomic analysis of renal allograft biopsies divided into three groups: no IFTA, mild IFTA or moderate to severe IFTA, and subsequently compared protein abundances between the groups. The authors identified 492 proteins uniquely expressed across the groups, and a further 904 proteins differentially expressed between the groups. Functional annotation revealed that proteins related to acute phase responses, actin cytoskeleton signaling and complement activation were particularly related to severe IFTA, which points towards immunologic factors playing a role in the progression of IFTA [12]. Two of the proteins identified in the current study, Coagulation factor XIII and Actin-related protein 2/3, belong to the same functional annotations identified by Nakorchevsky to be of importance in the development of IFTA.

In a proteomic analysis of renal allografts from rats, Reuter et al. identified ten proteins that were differentially regulated in allogeneic transplants compared to in syngeneic transplants. The authors pointed towards imbalances of energy homeostasis and oxidative stress as causal explanations for the identified proteins [24]. None of the identified proteins were found to correlate with the degree of fibrosis in the current study. These differences may be due to differences between the species, but additionally, the renal changes associated with the allogeneic rat transplantation model resemble chronic rejection [25] and thus may reflect more immune-driven processes than the present biopsies from stable renal transplant patients. Moreover, a study by Späth et al. confirmed our findings on the involvement of proteins related to inflammatory and fibrotic responses in kidney function and fibrosis [26].

Traditional evaluation of fibrosis is performed by semi-quantitative Banff-scoring in which the pathologist grades the extent of fibrosis as CI1 (<25%), CI2 (26%–50%) or CI3 (>50%) [27]. Since this scale is non-linear and allows for large variations within each category, a morphometric method (point counting) was chosen to quantify the extent of fibrosis in the current study. A previous study of point counting in renal allograft biopsies found a coefficient of variability of 7% with repeated measurements and established point counting as a reproducible way of quantifying interstitial fibrosis [28]. In renal allograft biopsies obtained 6 months post-transplantation, interstitial fibrosis evaluated by point counting was inversely correlated with renal function and predicted allograft survival [29]; however, no significant correlation was found in the current study. This is likely due to the limited sample size. Furthermore, sampling error may have influenced the results in some cases. Previous studies have similarly noted that there is not a strictly monotonous relationship between chronic allograft damage and renal function [5]. 

The proteome analysis does not discriminate between the renal compartments nor distinguish resident cells from invading cells. The morphometric evaluation of interstitial fibrosis aimed to provide an objective and continuous measure of the extent of fibrosis. The decision to include perivascular fibrosis served to minimize the bias introduced by subjective evaluation, but may have caused the overestimation of the extent of fibrosis, and thus weakened the correlation to renal function.

We are aware that the plethora of information yielded from the proteomic analysis poses the risk of giving false positive results. Moreover, it is well-known that the quantitative proteomic approach based on relative quantification using 10-plex TMT isobaric tags applied in the present study suffers from dynamic range compression that affects the linearity of the signal, which may also affect biological conclusions [30]. This underlines the need to interpret results in a biological context. In the current study, we have, however, performed corrections for multiple testing to keep the risk of type I error low. In summary, we have provided an extensive characterization of the renal proteome in stable renal transplant patients, and the suggestion of four novel biomarkers of renal allograft fibrosis. Our results support the notion that cytoskeleton organization and immune responses are prevalent processes in renal allograft fibrosis. The prognostic value of the identified biomarkers of fibrosis remains to be validated in independent cohorts. 

## 4. Methods

### 4.1. Participants

The study population included 31 patients from the SPIREN trial [31]. In brief, kidney transplant patients, at any time after the transplantation, were included from the outpatient clinic at Odense University Hospital. The eligibility criteria are listed in Table 5. At baseline, we performed a clinical examination, draw blood and urine samples, measured chrome-EDTA clearance, performed ambulatory blood pressure measurements, collected 24-hour urine samples and performed a kidney graft biopsy. 

The collection of kidney graft biopsies and laboratory variables, fibrosis quantification and ambulatory blood pressure measurements were done as previously described [28]. GFR was determined by chrome-EDTA clearance following the standard procedure, in the Department of Nuclear Medicine, Odense University Hospital, in which a single injection of ^51^CrEDTA was given, followed by the taking of a venous blood sample after 240 min to determine residual radioactivity. An additional blood sample 24 h after injection was taken from male patients with p-creatinine ≥200 µmol/L and female patients with p-creatinine ≥150 µmol/L. Ultrasound-guided kidney graft biopsies were performed by trained senior physicians in the Department of Nephrology, kept in formalin and subsequently embedded in paraffin. All biopsies were scored according to the Banff classification by an experienced renal pathologist. The Banff classification is a semi-quantitative score, where each biopsy is given a score between 0 and 3 for acute (tubulitis (T), interstitial mononuclear infiltration (I), glomerulitis (G), vascular (V) and arteriolar hyalinosis (AH)) or chronic changes (chronic glomerulopathy (CG), interstitial fibrosis (CI), tubular atrophy (CT) and chronic vascular changes (CV)) [32]. The morphometric quantification of fibrosis was determined by point counting performed on Masson Trichrome-stained sections using the Cast 2.0 software. In brief, this method estimates the volume fraction of fibrosis by superimposing a grid with 12 intersection points on a computerized image of the biopsy. After manually delineating the renal cortex, the program randomly selects sections of the biopsy for quantification. The volume fraction of fibrosis is calculated by determining the number of intersection points that overlie fibrotic areas relative to the number of intersection points overlying normal renal tissue. In the current study, no exclusion of perivascular fibrosis was performed.

### 4.2. Proteomics

#### 4.2.1. Sample Preparation

Sections of formalin-fixed, paraffin-embedded kidney biopsies were washed three times in chloroform. The proteins were extracted by dissolving the deparaffinized tissue sections in extraction buffer (1M dithiothretiol (DTT), 0.2 M tetraethylammonium bicarbonate (TEAB) and 10% sodium dodecyl sulphate (SDS)) followed by two rounds of ultrasonification (15 min of ultrasonification/15 min of cooling on ice), and incubated at 99 °C for 20 min then at 80 °C for 120 min. Protein alkylation was done by adding a 200 mM iodoacetamide (IAA) solution to a final DTT/IAA concentration ratio of 1:3. The acetone-precipitated proteins were re-dissolved in 5 µL 8 M urea with 1 µg LysC and incubated at 30 °C for 4 h, followed by a further dilution to 1 M urea, the addition of 2 µg trypsin, and an overnight incubation at 30 ˚C. The resulting tryptic peptides were isotopically labelled using the 10-plex tandem mass tag (TMT, ThermoFisher Scientific, Waltham, MA, USA). Peptide samples were randomly labelled with the 127N, 127C, 128N, 128C, 129N, 129C and 130N mass tags, whereas a pool of all samples were labelled with the mass tag 126 that served as an internal standard. Tagged peptides were mixed into 7 mixed peptide samples that were fractionated using hydrophilic interaction chromatography (HILIC), as described below.

#### 4.2.2. HILIC Fractionation

Briefly, the labelled peptide mixtures were re-dissolved in 90% ACN/0.1% TFA, and 15 µL aliquots corresponding to approximately 25 µg peptides were injected onto an in-house-packed TSK gel Amide-80 HILIC 300 μm × 300 mm capillary high-performance liquid chromatography (HPLC) column and fractionated into 42 fractions by a Dionex UltiMate 3000 nanoHPLC using a linear 59 min gradient (85.5% B to 54% B; solvent A: 0.1%TFA; solvent B: 90% ACN/0.1% TFA) at a flow rate of 6 µL per minute. The fractions were automatically collected in micro-well plates at 1-minute intervals after UV detection at 210 nm. The fractions were dried by vacuum centrifugation, re-dissolved in 10 µL 0.1% TFA and analyzed by nano-LC–MS/MS, as described below.

#### 4.2.3. NanoLC-MS/MS

A Q-Exactive mass spectrometer (Thermo Fisher Scientific, Bremen, Germany) equipped with a nano-HPLC interface (Dionex UltiMate 3000 nano HPLC) was applied for nano-LC-MSMS analyses. The samples (5 µL) were loaded onto a custom-made, fused capillary pre-column (2 cm length, 360 µm OD, 75 µm ID) and separated on a custom-made fused capillary column (20 cm length, 360 µm OD, 100 µm ID—both columns were packed with ReporSil Pur C13 3 µm resin) using a linear gradient from 95% solution A (0.1% formic acid) to 30% B (100% acetonitrile in 0.1% formic acid) over 51 min, followed by 5 min at 90% B and 5 min at 98% A, at a flow rate of 300 nL per minute. The acquisition of mass spectra was done in positive ion mode using an automatic data-dependent switch between an Orbitrap survey MS scan in the mass range from 400 to 1200 m/z, and high-energy collisional dissociation fragmentation (HCD) and Orbitrap detection of the 15 most intense ions observed in the MS scan. The Orbitrap target values for the MS and MSMS scans were 1,000,000 and 50,000 ions, at resolutions of 70,000 and 35,000 at m/z 200. Fragmentation in the HCD cell was performed at the normalized collision energy of peptides and 31 eV for TMT-labelled peptides. The ion selection threshold was set to 17,000 counts. Selected sequenced ions were dynamically excluded for 60 s. The data are available via ProteomeXchange with the identifier PXD017867. Reviewer account details; Username: reviewer23512@ebi.ac.uk; password: AOuDknvS.

### 4.3. Data Analysis

The Sequest search engine and Mascot search Engine (v. 2.2.3) integrated with the Proteome Discoverer (PD) version 2.1 software (Thermo Scientific) were used to search raw data files with the following criteria; Protein database: Uniprot/Swissprot (downloaded 7th November 2012, 452,768 entries) and restricted to humans. Trypsin, one missed cleavage allowed, carbamidomethylation at cysteines, and 10-plex TMT labelling at lysine and N-terminal amines were set as fixed parameters, while methionine oxidation and deamidation were set as dynamic. The precursor mass tolerance was set to 8 PPM and the MSMS tolerance was set to 0.05 Da. The peptide data were extracted using a Mascot significance threshold of 0.05 and a minimum peptide length of 6. The false discovery rate (FDR) was calculated using a decoy database search, and only high-confidence peptide identifications (false discovery rate <1%) were included. The data were normalized by using the “Total peptide amount” setting in the Reporter Ions Quantifier node in PD, i.e., the sum of abundance values for each channel over all peptides identified within a file was calculated, and the all channels were normalized against the channel with the highest total abundance. Search files were further processed using the Proteome Discoverer software. Gene Ontology analysis was performed using the GOrila tool for GO enrichment analysis (http://cbl-gorilla.cs.technion.ac.il/GOrilla/kh37217t/GOResults.html) [33].

### 4.4. Statistical Analysis

Analyses of baseline values were performed using Stata15 (StataCorp, College Station, TX, USA). The data are described by the median (interquartile range). Due to the non-Normal distribution of the fibrosis data, a non-parametric correlation test was used (Spearman’s rank correlation). The association of specific proteins with fibrosis was evaluated using the R software. For each protein, we applied a multiple linear regression model for the volume fraction of fibrosis, with sex, age and donor type (deceased/living related) as covariates. The resulting *p*-values were corrected for multiple testing by the Benjamini-Hochberg procedure. To find a subset of proteins predicting the degree of fibrosis, we used LASSO with stability selection [34]. This method identifies the proteins with the highest predictive value with regards to fibrosis. The LASSO was applied to a linear model where all proteins with no missing values (1973 proteins), sex, age, donor type, and time since transplantation were used as covariates. This was repeated 1000 times on a randomly selected subsample consisting of 16 samples. The probability of selecting each protein was computed. 

### 4.5. Ethics

All subjects provided written consent to participate in the study. The project was performed in accordance with the International Conference of Harmonization—Good Clinical Practice (ICH—GCP) guidelines and has been approved by the Danish Research Ethics Committee and the Danish Data Protection Agency. The SPIREN trial was registered in ClinicalTrials.gov (05/17/2012): NCT01602861, EudraCT (05/31/2011): 2011-002243-98.

## Figures and Tables

**Table 1:** The clinical characteristics of the study population (*n* = 31). The data are presented as medians (interquartile range).

Item	Value
Male sex	22 (71%)
Body Mass Index (kg/m^2^)	26 (24–28)
Age (years)	60 (50–66)
Smoking (never/previous/current)	16/9/6 (52%/29%/19%)
Age of current transplant (years)	3.0 (1.6–9.5)
Donor type	
Living related	12
Deceased	19
Previous dialysis	24 (77%)
Previous rejection	3 (10%)
Borderline	1 (3%)
Grade 1A	2 (6%)
Cause of renal failure	
Hypertension	6 (19%)
Diabetes	2 (6%)
Polycystic kidney disease	9 (29%)
IgA nephritis	2 (6%)
Glomerulonepritis	3 (10%)
Congenital malformation	3 (10%)
Reflux nefropathy	1 (3%)
Unknown	5 (16%)
Comorbidity	
Hypertension	29 (94%)
Diabetes	2 (6%)
Apoplexia	1 (3%)
Myocardial infarction	3 (10%)
Heart failure	0 (0%)
Current medication	
ACE-inhibitors	14 (45%)
Angiotensin II receptor blockers	2 (6%)
Calcium channel antagonists	18 (58%)
β-adrenergic antagonists	17 (55%)
α-adrenergic antagonists	5 (16%)
Loop diuretics	4 (13%)
Thiazide diuretics	2 (6%)
Cholesterol lowering drugs	4 (13%)
Calcineurin inhibition:	
-Tacrolimus	25 (81%)
-Cyclosporine A	6 (19%)
Antimetabolite:	
-Mycophenolate mofetil	29 (94%)
-Azathioprine	2 (6%)
Prednisolone	4 (13%)
Systolic blood pressure (mmHg)	131 (118–141)
Diastolic blood pressure (mmHg)	79 (75–83)
Chrome-EDTA clearance (mL/min)	49 (39–60)
Fibrosis (%)	33 (30–41)

**Table 2:** Proteins with FDR < 0.5 multiple linear regressions after correction for multiple testing.

Protein	Accesion Number ^A^	Estimate ^B^	SE ^C^	*p*-Value	Adjusted *p*-Value ^D^	Missing values ^E^
Uridine phosphorylase 1	Q16831	19.4	2.5	3.06 × 10^-7^	8.21 × 10^-4^	8
Cytochrome c oxidase assembly factor 6 homolog	Q5JTJ3.2	−44.9	6.9	6,98 × 10^-7^	1.88 × 10^-3^	0
Coagulation factor XIII A chain	P00488	18.7	3.4	9.09 × 10^-6^	2.44 × 10^-2^	0
Actin-related protein 2/3 complex subunit 2	O15144	34.3	6.5	1.78 × 10^-5^	4.77 × 10^-2^	0
WD repeat-containing protein 82	Q6UXN9	34.8	6.0	3.38 × 10^-5^	9.08 × 10^-2^	11
Actin-related protein 2/3 complex subunit 1B	O15143	21.6	4.5	5.17 × 10^-5^	1.39 × 10^-1^	0
Heterogeneous nuclear ribonucleoprotein U	Q00839	48.3	10.0	5.38 × 10^-5^	1.44 × 10^-1^	0
Mitochondrial aconitate hydratase	A2A274	−47.2	10.1	8.20 × 10^-5^	2.20 × 10^-1^	0
Hematopoietic lineage cell-specific protein	P14317	11.3	2.5	9.49 × 10^-5^	2.54 × 10^-1^	0
Lamina-associated polypeptide 2, isoforms beta/gamma	P42167	25.7	5.6	1.06 × 10^-4^	2.84 × 10^-1^	0
NADH dehydrogenase 1 alpha subcomplex subunit 2	O43678	−38.4	8.5	1.13 × 10^-4^	3.01 × 10^-1^	0
60S ribosomal protein L7a	P62424	56.2	12.4	1.17 × 10^-4^	3.14 × 10^-1^	0
Cathepsin G	P08311	9.9	2.2	1.18 × 10^-4^	3.16 × 10^-1^	0
Perilipin-3	O60664	33.6	7.3	1.21 × 10^-4^	3.24 × 10^-1^	3
Dimethylglycine dehydrogenase, mitochondrial	Q9UI17	−26.6	5.9	1.22 × 10^-4^	3.26 × 10^-1^	0
60S ribosomal protein L5	P46777	36.3	8.1	1.28 × 10^-4^	3.43 × 10^-1^	0
Proteasome subunit beta type-8	P28062	26.8	6.0	1.35 × 10^-4^	3.61 × 10^-1^	0
HLA class I histocompatibility antigen, B-42 alpha chain	P30480	17.6	3.9	1.37 × 10^-4^	3.65 × 10^-1^	0
Xaa-Pro aminopeptidase 2	O43895	−27.5	6.2	1.41 × 10^-4^	3.78 × 10^-1^	0
Coronin-1A	P31146	5.9	1.3	1.80 × 10^-4^	3.94 × 10^-1^	0
Tubulin beta chain	P07437	36.3	8.2	1.54 × 10^-4^	4.10 × 10^-1^	0
Splicing factor U2AF 65 kDa subunit	P26368	25.7	5.8	1.54 × 10^-4^	4.11 × 10^-1^	0
Short/branched chain specific acyl-CoA dehydrogenase, mitochondrial	P45954	−26.9	6.1	1.58 × 10^-4^	4.20 × 10^-1^	0
X-ray repair cross-complementing protein 6	P12956	40.2	9.1	1.63 × 10^-4^	4.33 × 10^-1^	0
Delta(3,5)-Delta(2,4)-dienoyl-CoA isomerase, mitochondrial	Q13011	−37.2	8.5	1.72 × 10^-4^	4.58 × 10^-1^	0
Sodium-dependent neutral amino acid transporter B(0)AT1	Q695T7	−21.4	4.9	1.79 × 10^-4^	4.77 × 10^-1^	0

**^A^**: Swissprot accession number; **^B^**: Estimated regression coefficient; **^C^**: Standard Error; **^D^**: Values are corrected according to the Benjamin-Hochberg procedure; **^E^**: The number of missing values for the specific protein out of the study population of 31 patients.

**Table 3:** Proteins chosen by the LASSO in more than 10% of all subsamples.

Protein	Accession Number	Correlation	Biological Process	Selection Probability
Cytochrome c oxidase assembly factor 6 homolog	Q5JTJ3.2	Negative	Cell respiration	0.567
Apoptosis inhibitor 5	Q9BZZ5.2	Positive	Inhibition of (fibroblast) apoptosis	0.234
Coagulation factor XIII A chain	P00488	Positive	Coagulation/cross-linking ECM proteins/inhibition of fibroblast apoptosis	0.217
Microtubule-associated protein 1B	P46821	Positive	Neuronal cell structure maintainance	0.205
Splicing factor U2AF 65 kDa subunit	P26368	Positive	mRNA processing	0.199
Symplekin	Q92797	Positive	Cell adhesion/mRNA processing	0.156
Threonine synthase-like 1	Q8IYQ7	Negative	Unknown	0.131
AP-2 complex subunit sigma	P53680	Positive	Protein transport	0.118
Actin-related protein 2/3 complex subunit 2	O15144	Positive	Actin cytoskeleton organization	0.107

**Table 4:** Significantly enriched Gene Ontology categories. Gene ontology (GO) enrichment analysis of proteins that are significantly (*p* < 0.005) positively and negatively associated with fibrosis as determined by multiple regression analysis (Supplementary Table S4). GO categories were identified using two unranked list options of the GOrilla Gene Ontology (GO) program (http://cbl-gorilla.cs.technion.ac.il/). N is the total number of recognized proteins, B is the total number of recognized proteins associated with a specific GO term, n is the number of recognized proteins in the target set, and b is the number of proteins in the intersection. Enrichment = (b/n)/(B/N). The *p*-Value is the enrichment *p*-value computed according to the mHG or HG model. This *p*-value is not corrected for the multiple testing of 2893 GO terms, and the FDR *q*-value is the correction of the above *p*-value for multiple testing using the Benjamini and Hochberg method.

GO Term	Description	*p*-Value	FDR *q*-Value	Enrichment (N, B, n, b)
*Categories positively associated with fibrosis*				
GO:0010468	regulation of gene expression	2.09 × 10^-7^	1.97 × 10^-3^	1.89 (2240,546,113,52)
GO:0051704	multi-organism process	2.37 × 10^-7^	1.11 × 10^-3^	2.22 (2240, 348, 113, 39)
GO:0050789	regulation of biological process	5.94 × 10^-7^	1.86 × 10^-3^	1.34 (2240, 1371, 113, 93)
GO:0010629	negative regulation of gene expression	6.29 × 10^-7^	1.48 × 10^-3^	2.47 (2240, 249, 113, 31)
GO:0044419	interspecies interaction between organisms	8.74 × 10^-7^	1.64 × 10^-3^	2.38 (2240, 266, 113, 32)
GO:0002376	immune system process	1.27 × 10^-6^	1.99 × 10^-3^	1.96 (2240, 446, 113, 44)
GO:0002682	regulation of immune system process	1.41 × 10^-6^	1.90 × 10^-3^	2.38 (2240, 258, 113, 31)
GO:0045321	leukocyte activation	1.46 × 10^-6^	1.72 × 10^-3^	2.43 (2240, 245, 113, 30)
GO:0006952	defense response	1.83 × 10^-6^	1.91 × 10^-3^	2.87 (2240, 159, 113, 23)
GO:0002252	immune effector process	3.34 × 10^-6^	3.14 × 10^-3^	2.21 (2240, 296, 113, 33)
GO:0048518	positive regulation of biological process	3.65 × 10^-6^	3.12 × 10^-3^	1.55 (2240, 844, 113, 66)
GO:0006955	immune response	5.25 × 10^-6^	4.11 × 10^-3^	2.98 (2240, 133, 113, 20)
GO:0050794	regulation of cellular process	5.83 × 10^-6^	4.21 × 10^-3^	1.36 (2240, 1239, 113, 85)
GO:0007166	cell surface receptor signaling pathway	8.47 × 10^-6^	5.68 × 10^-3^	2.16 (2240, 294, 113, 32)
GO:0001775	cell activation	9.28 × 10^-6^	5.81 × 10^-3^	2.23 (2240, 267, 113, 30)
GO:0002684	positive regulation of immune system process	9.39 × 10^-6^	5.52 × 10^-3^	2.54 (2240, 187, 113, 24)
*Categories negatively associated with fibrosis*				
GO:0055114	oxidation-reduction process	1.94 × 10-15	1.79 × 10^-11^	3.75 (2198, 318, 70, 38)
GO:0019752	carboxylic acid metabolic process	1.16 × 10-11	5.37 × 10^-8^	3.16 (2198, 348, 70, 35)
GO:0043436	oxoacid metabolic process	4.53 × 10-11	1.39 × 10^-7^	3.02 (2198, 364, 70, 38)
GO:0044281	small molecule metabolic process	6.03 × 10-11	1.39 × 10^-7^	2.40 (2198, 576, 70, 44)
GO:0006082	organic acid metabolic process	8.68 × 10-11	1.60 × 10^-7^	2.95 (2198, 372, 70, 35)

**Table 5:** The eligibility criteria.

Inclusion Criteria	Exclusion Criteria
Age > 18 years	Former intolerance of spironolactone
Tacrolimus/cyclosporine treatment	Potassium binder or digoxin treatment
Proteinuria < 3 g/day	Pregnancy or planned pregnancy
Creatinine clearance ≥ 30 mL/min	Clinically relevant organic, systemic or psychological disorder
Plasma potassium < 5.5 mmol/L	Expectation of non-compliance
Negative pregnancy test at inclusion for women of childbearing potential and adequate contraception throughout the trial	

## Supplementary Materials

Supplementary materials can be found at https://www.mdpi.com/1422-0067/21/7/2371/s1.
